# Management of optic neuritis

**DOI:** 10.4103/0301-4738.77020

**Published:** 2011

**Authors:** Vimla Menon, Rohit Saxena, Ruby Misra, Swati Phuljhele

**Affiliations:** Dr. R. P. Centre for Ophthalmic Sciences, All India Institute of Medical Sciences, New Delhi, India

**Keywords:** Disc edema, multiple sclerosis, optic neuritis, papillitis

## Abstract

Optic neuritis is an inflammatory condition of the optic nerve characterized by a sudden onset of unilateral visual loss, usually affecting young females. Demyelination associated with multiple sclerosis (MS) is the most common cause in regions where MS is prevalent; while in other places, there are a substantial proportion of cases where infective or autoimmune causes are seen. Optic Neuritis Treatment Trial (ONTT) was the first major study that provided information on the natural history, role of steroids in treatment and risk of development of MS. Subsequently, numerous clinical trials have evaluated different modalities of management of optic neuritis and MS. The Controlled High-Risk Subjects Avonex Multiple Sclerosis Prevention Study (CHAMPS); the Prevention of Relapses and Disability by Interferon β-1a Subcutaneously in Multiple Sclerosis (PRISMS) Trial; and, most recently, the Betaferon in Newly Emerging Multiple Sclerosis for Initial Treatment (BENEFIT) Study have provided large amount of information on the natural history of optic neuritis and management options available. However, due to the low prevalence of MS reported in Asian studies, high cost of therapy and indefinite time period of treatment, it may not be cost effective to start interferon therapy in most cases.

Optic neuritis is an inflammatory condition affecting the optic nerve, usually affecting young adults, especially females, between 18 and 45 years of age. Although it has been reported from almost all parts of the world, regions with the highest incidence include northern Europe, southern Australia and middle part of North America.[1,2] Most of the cases are idiopathic in nature; however, it could be associated with demyelinating lesions, of which multiple sclerosis (MS) is the most common cause. Other less common etiologies include infectious and para-infectious causes, inflammatory and para vaccination immunological responses [Fig. 1].

**Figure 1:**
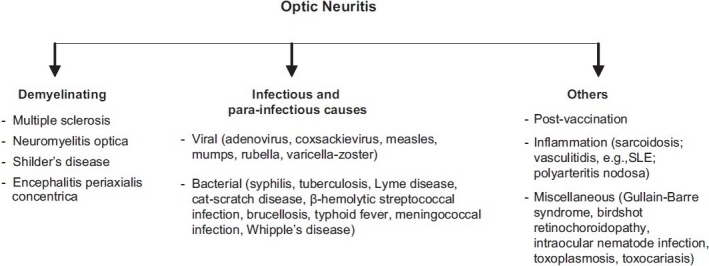
Causes of optic neuritis

## Clinical Features

Typical optic neuritis in adults usually presents as acute monocular loss of vision progressing over several hours to days, often associated with ocular pain that worsens on eye movement. Presenting vision can range from 20/20 with mild visual defects to no light perception. The Optic Neuritis Treatment Trial (ONTT) reported a vision of 20/20 or better at presentation in 10.5% of the patients of optic neuritis, and no perception of light in 3.1% of the cases.[3] Although vision loss is usually monocular, involvement of both eyes can occur, usually in children.[4] A relative afferent pupillary defect is present in almost all unilateral cases, but absence of the defect suggests a pre-existing or coincident optic neuropathy in the fellow eye. Although retrobulbar optic neuritis (normal disc appearance) is more common,[3] quite a substantial proportion of patients have disc edema (papillitis), particularly in this part of the world.[5,6] A variety of visual field defects ranging from commonly seen diffuse depression and centrocecal scotoma to rarely seen quadrantic defects and altitudinal defects are present in patients with optic neuritis. Subnormal color vision or contrast sensitivity is noted in the affected eye and at times in the fellow eye and is suggestive of subclinical involvement of the fellow eye. Recovery of vision usually begins within the first month. In the ONTT, 79% of the participants had started to improve by 3 weeks; and 93%, by 5 weeks.[7] Improvement may continue after this, especially in patients with poor vision, up to a period of 12 months. Though good functional visual recovery is seen in most patients, around 5% to 10% of patients fail to recover fully. Atypical features include absence of pain, which may be seen in only 8% of the patients with typical optic neuritis; marked swelling of the nerve with retinal exudates and peripapillary hemorrhages; severe visual loss to no light perception; progression of visual loss or pain for more than 2 weeks; and lack of recovery after 3 weeks.[8,9] Bilateral optic neuritis may occur, either simultaneously or sequentially,[10] which would also be an unusual feature in typical optic neuritis.[11] Patients with atypical optic neuritis are at lower risk of developing MS and should be extensively evaluated for other causes of optic neuropathy.

Asian MS has traditionally been thought of as a distinct entity different from that seen in the west, characterized by high incidence of visual involvement at onset, prevalence of recurrent acute transverse myelitis [considered as an optico-spinal MS (OSMS)] and a high degree of overlap with neuron myelitis optica (NMO). [12,13] In a prospective study done in Chandigarh by Jain *et al*.,[14] 42 patients of optic neuritis were followed up for a period up to 6 months. In 20 (29.4%) eyes, the appearance of the optic disc was normal, indicating retrobulbar neuritis; whereas 38 (56%) eyes showed blurring of the disc margins with or without edema of the disc, suggestive of papillitis or anterior retrobulbar neuritis. Sixty-two percent had bilateral involvement of the discs. Only 3 (7.1%) patients had some neurological deficit, and a provisional diagnosis of MS was made. Similar clinical profile has been reported in the other studies from Asian region.[5,6,15,16] Higher incidence of involvement of optic nerve head, less incidence of pain, less brain magnetic resonance imaging (MRI) abnormalities, severe visual loss and poorer visual outcome are common findings among the population from Asia. The incidence of MS reported in these studies was much lower than that reported in the western literature. In a retrospective study of Taiwanese patients with acute optic neuritis,[17] it was found that the 5-year cumulative probability of conversion to MS was 14.28%.

## Epidemiology

Region-wise estimates of the incidence of demyelinating optic neuritis are unavailable. Optic neuritis is reported to have an incidence of 1-5 cases per 100,000/year; higher the latitude, higher was found to be the incidence of optic neuritis.[18–20] While the estimated prevalence of MS in the United States and England is 46 per 100,000 and 93 per 100,000, respectively,[2] the prevalence in eastern countries varies from 0.77 to 1.8 per 100,000,[21,22] suggesting that the profile of optic neuritis patients is different in the eastern and western parts of the world. The optico-spinal variant of MS, which is characterized by involvement of only the optic nerves and spinal cord with no brain lesions, is more prevalent in this part of the world and can be easily misdiagnosed as NMO, which again is widespread in this region.[13,23] Pandit *et al*.[24] found 47% of their MS cases to have clinical attacks confined to the optic nerve and spinal cord. There are no large-scale epidemiological studies from India on the incidence and prevalence of MS. Indian studies have shown that MS constitutes 0.32% to 1.58% of neurology admissions in hospitals,[25–28] and a prevalence of approximately 1.33/100,000 was reported by Singhal *et al*. in the mid-eighties from the west coast of India.[29] However, the incidence of NMO has been reported to be 9.5% in a recent study in India.[24]

## Diagnosis / Ancillary Testing and Management [Fig. 2]

**Figure 2:**
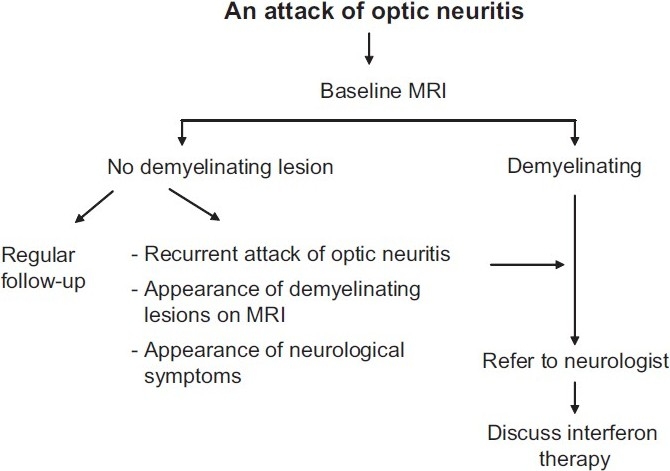
Management protocol for patients of optic neuritis

The diagnosis of optic neuritis is a clinical one, made in a patient of appropriate age with the clinical features mentioned above. Although no investigations are necessary for confirming the diagnosis, investigations are needed to assess the risk of developing MS and to rule out other disorders. In atypical cases, e.g., those of patients older than 50 years, or cases with a history suggestive of a secondary etiology, an additional workup to identify the etiology should be considered. Since tuberculosis is common in our country, a baseline chest x-ray is done in all patients prior to starting treatment with steroids. Serology for syphilis, bartonella and toxoplasmosis; Mantoux test; blood culture; cerebrospinal fluid (CSF) examination; and blood tests should be done to rule out infective and inflammatory cases. The choice of investigations depends upon the clinical picture, for example, MRI of the optic nerves in suspected cases of compressive optic neuropathy; computerized tomography (CT) of the orbits in bony orbital lesions; orbital ultrasound for posterior scleritis; optical coherence tomography, fluorescein angiography, and electroretinography for retinal diseases. Specialized tests, e.g., toxin screens and serum B12 for toxic optic neuropathy; markers for autoimmune diseases; antibody to aquaporin-4 and MRI spine in NMO; genetic analysis for mitochondrial mutation in cases of Leber’s hereditary optic neuropathy, are required in suspected conditions.

Though MacDonald’s criteria recommend MRI of brain and spinal cord to diagnose MS, the diagnosis of MS in India has relied heavily on clinical criteria owing to lack of facilities and/ or financial constraints in the general population. Radiological and other paraclinical tests are not afforded by all patients. In addition to the above facts, the clinical presentation of MS in the form of OSMS, which is similar to that of NMO, confuses the scenario.

### Recommendations for MRI

In populations with a high incidence of MS, all patients with acute monosymptomatic demyelinating optic neuritis should undergo gadolinium-enhanced MRI of the brain and spine to determine if they are at a high risk for the subsequent development of clinically definite multiple sclerosis (CDMS).[30] MRI imaging becomes essential in recurrent optic neuritis, in patients keen to know about long-term prognosis, in patients with a history or evidence of other neurological involvement, in atypical cases[30] and in acute optic neuritis in children.[31] The presence of demyelinating lesions on brain MRI at the time of clinical presentation is the strongest predictor for developing CDMS. MRI showing ≥2 white matter lesions (≥3 mm in diameter, at least 1 lesion periventricular or ovoid) indicates a high risk for CDMS.[32,33]

On follow-up of the patients of the ONTT group after a period of 15 years, it was seen that the cumulative probability for developing MS after the first episode of optic neuritis was 50%, the risk being highest in the first 5 years. If the MRI was negative at baseline, the overall risk of MS was 25%. Presence of a single lesion doubled the 15-year risk to 50%, while three or more lesions caused a threefold increase in the risk, viz., to 78%.[34]

### Role of steroids

The most reliable information concerning long-term outcome of optic neuritis comes from the ONTT,[35] which was a multi-centered randomized trial involving 454 patients from 1988 to 2006, mainly undertaken to evaluate the efficacy of corticosteroid treatment for acute optic neuritis and to investigate the relationship between optic neuritis and MS. The cases included in the study had a clinical picture consistent with unilateral optic neuritis, visual loss lasting 8 days or less with no previous episodes of optic neuritis in the affected eye, no previous steroid treatment for MS or optic neuritis and no other systemic disease except MS as a cause of optic neuritis. The patients were randomized into 3 different treatment groups: oral placebo for 14 days; intravenous methylprednisolone (250 mg, 6 hourly) for 3 days, followed by oral prednisolone (1 mg\kg\d) for 11 days and a 3-day tapering of prednisolone thereafter; oral prednisolone (1 mg\kg\d) for 14 days, followed by a 3-day tapering.

The results showed that the intravenous steroid group recovered vision faster compared to those treated with oral administration or placebo, but the difference in the rate of recovery subsided within 1 month. Intravenous steroids afforded a short-term but statistically significant benefit in contrast sensitivity, color vision and visual field but not in visual acuity at 6 months. At the 1-year follow-up, there was no statistically significant difference in visual function among the groups. Visual acuity was 20/ 40 or better in 95% of the placebo group, 94% of the intravenous steroid group and 91% of the oral steroid group at 1 year.[36] After15 years, 72% of the eyes affected with optic neuritis had visual acuity of ≥ 20/20, and 66% of the patients had ≥ 20/20 acuity in both eyes.[37]

The interesting finding was that patients with oral regimen had a twofold greater rate of recurrent optic neuritis. Of the patients treated with oral steroids, 30% experienced a recurrence, either in the fellow eye or in the affected eye in the first 2 years of follow-up, compared to only 13% in the intravenous steroid group and 16% in the placebo group.[38] In the first 2 years of follow-up, intravenous steroids reduced the risk of developing MS compared to the other 2 groups. At 2 years, 8% of the patients treated with intravenous steroids had clinically definite MS, whereas 18% of the placebo group and 16% of the oral steroid group developed MS. Among the patients who had not developed multiple sclerosis at 5 years after study enrollment, the probability of being diagnosed as having MS between 5 and 10 years was 7% in 142 patients with no lesions on MRI and 27% in the 89 patients with 1 or more lesions.

However, it should be noted that the baseline MRI scans in this study were performed before availability of gadolinium enhancement and advanced inversion recovery imaging. The overall high rate of 32% for conversion to MS between years 10 and 15 suggests that patients should be followed up for a long period of time. Among the patients who had no lesions on MRI, it was found that male gender and optic disc swelling were associated with a lower risk of MS, as was the presence of the following atypical features: no light perception vision; absence of pain; and ophthalmoscopic findings of severe optic disc edema, peripapillary hemorrhages, or retinal exudates.

Based on the findings of the ONTT, the study group made several recommendations:[39]

- Chest x-ray, blood tests and lumbar puncture are not indicated for typical cases of optic neuritis
- Treatment with oral prednisolone in conventional doses alone, is contraindicated
- Consider treatment with intravenous steroids when 3 or more signal abnormalities are present on MRI to reduce 2-year risk of developing MS, or in patients requiring expedited recovery of vision (i.e., monocular patients, employment demands, bilateral involvement and patients desiring intervention).

Studies with higher doses of oral corticosteroids (methylprednisolone) *vs*. placebo have been conducted, but no statistically significant benefit could be seen on a long-term basis or in the relapse rate.[40] An observational study conducted at our center to evaluate the efficacy and safety profile of intravenous dexamethasone showed that the intravenous pulse dexamethasone led to rapid recovery of vision in acute optic neuritis, without any serious side effects.[41] Later on, a case-control study[42] was done to compare the efficacies of intravenous mega-dose methylprednisolone and intravenous dexamethasone in terms of visual recovery, as well as to evaluate their side effects. Intravenous dexamethasone was found to be as effective as mega-dose intravenous methylprednisolone therapy recommended by the ONTT study, with the added advantage of being easier to administer and less costly (costing one sixth of injection methylprednisolone). At our center, we routinely use 200 mg intravenous dexamethasone for 3 days, followed by oral prednisolone.

A recent study[43] regarding the use of intravenous methylprednisolone given at regular intervals has been completed. A single monthly infusion of 500 mg methylprednisolone with a 3-day oral tapering can reduce inflammatory disease activity in patients with relapsing-remitting MS, without clinically relevant side effects. There was a reduction in the number of gadolinium-enhanced lesions and T2 lesion load over a 6-month follow-up period.

### Role of immunomodulators

At present the immunomodulating drugs that have been shown to reduce the development and severity of CDMS include interferon β-1a (Avonex^®^, Rebif^®^), interferon β-1b (Betaseron^®^) and Glatiramer acetate.[44] The various mechanisms proposed include reduced antigen presentation, inhibition of pro-inhibitory cytokines and autoreactive T cells, induction of immunosuppressive cytokines and decreased migration of cells in the central nervous system (CNS). Another antineoplastic, immunomodulatory agent, viz., Mitoxantrone (Novantrone^®^),[45] a synthetic anthracenedione derivative, when given intravenously has been shown to improve neurological disability and result in delayed progression of MS in patients with worsening relapsing-remitting (RR) or secondary-progressive (SP) disease.

CHAMPS (Controlled High-Risk Subjects Avonex Multiple Sclerosis Prevention Study)[46] was a randomized, double-blind trial involving 383 patients with an initial, acute monosymptomatic demyelinating event (unilateral optic neuritis, incomplete transverse myelitis, or brainstem/ cerebellar) and at least 2 silent T2 lesions on brain MRI. The patients were randomized to weekly intramuscular interferon-β1a (Avonex®, Biogen Idec) or placebo. The treatment group experienced a 44% reduction in the rate of development of CDMS compared with the placebo group over 3 years of follow-up. There were statistically significant beneficial effects on all MRI parameters for the treatment group, including decrease in T2 lesion development, gadolinium-enhancing lesions and T2 lesion volume.

The 10-year follow-up showed that patients treated immediately after their first episode had a significantly lesser chance of experiencing a second attack compared to those who had delayed treatment (after about 30 months). This showed the advantages of initiating early treatment with interferon-β1a.

The most common side effects associated with Avonex are flu-like symptoms, including myalgia, fever, fatigue, headache, chills, nausea, vomiting, pain and asthenia.[47]

The PRISMS[48] (Prevention of Relapses and Disability by Interferon β-1a Subcutaneously in Multiple Sclerosis) Trial assessed efficacy of interferon (IFN)-β1a in dosages of 22 µg and 44 µg s.c. given subcutaneously compared to placebo in RRMS (relapsing-remitting multiple sclerosis) patients, and it was seen that both the treatment groups had fewer relapses.

The most recent study has been the Betaferon in Newly Emerging Multiple Sclerosis for Initial Treatment (BENEFIT) study, which included patients with a single neurologic event and at least 2 clinically silent MRI lesions. In a 24-month study period, standard dose of interferon-β1b (Betaseron^®^, Bayer Health Care Pharmaceuticals) was seen to reduce the risk of MS by 50%.[49]

Similarly, intravenous immunoglobulin treatment tried recently has been found to reduce the rate of conversion to MS and decrease accumulation of several MRI abnormalities over a 1-year period after CIS (clinically isolated syndrome).[50]

Our current approach regarding initiation of immunomodulator therapy is based on the following facts: (1) low prevalence of MS in Asian countries; (2) expense of Rs. 6,000 per injection for Avonex ^®^, which would amount to Rs. 312,000 per year; (3) no end point to the duration of the treatment; (4) disease-modifying drugs are only partially effective in the short-term; besides, prevention of disability in the long-term is unproven; moreover, with the prolonged treatment, it is hard to distinguish whether a favorable outcome reflects a favorable natural history or successful treatment in an individual patient, especially if treatment is started without a period of observation; (5) MS often has a favorable natural history.

Patients who have a normal MRI of the brain are kept under regular follow-up. These patients should undergo surveillance MRI (at least annually at first) to look for the development of white matter lesions, as the ONTT showed even this cohort has a 22% risk of developing MS. Patients with 1 or more lesions are counseled regarding the pros and cons of immunomodulator therapy and kept under close follow-up with serial monitoring of MRI scans [Fig. 1].

We avoid treating those with a greater chance of benign course (i.e., those with a low expanded disability status scale (EDSS)[51] score at 5 years and/ or a low attack rate and little accumulation of MRI lesions early in the disease course), a low chance of benefit (i.e., patients with established progressive disease without clinical or radiological markers of ongoing inflammatory disease); and those with an indeterminate prognosis (e.g., CIS or early relapsing-remitting MS with infrequent mild attacks and a favorable prognostic profile).

The risk of MS is much lower in children than in adults. One large, retrospective study found the cumulative risk of developing MS (the study predated the McDonald criteria) was 13% at 10 years and 19% by 20 years.[52] Besides, use of immunomodulatory therapies to reduce the risk of MS has not been well studied in children. Hence children with optic neuritis, most often bilateral and of viral etiology, are treated with intravenous steroids.

## Prognosis

The long-term visual prognosis of idiopathic optic neuritis remains good. More than 90% of the patients recover a visual acuity of 20/40 or better by 6 months, as seen in the ONTT. A cut-off level of vision ≤ 20/50 (6/15), contrast sensitivity of <1.0 log units and a visual field mean deviation of ≤ – 15 dB after 1 month in the ONTT were predictive of a poor visual outcome at 6 months, a prediction that could not be made with any certainty at baseline.[53] On the other hand, the OSMS form of MS that is prevalent in the Asian region is associated with poor visual recovery.[54] However, it should be kept in mind that the other causes of optic neuritis, specifically NMO and infective, which are more prevalent in this region, may skew the data regarding the outcome of optic neuritis.

Despite the relatively good visual outcome, most patients show a degree of long-lasting damage to the optic nerve, indicated by a pale optic disc, loss of retinal nerve fibers, prolonged latency in the visual evoked response and a thinning of the optic nerve on MRI. Patients who have had an attack of optic neuritis are at a risk of recurrence, with at least one documented recurrence in either one or both eyes being 35% in 10 years (ONTT study). This risk was twice as high in those who eventually developed MS (48% *vs*. 24%; *P*< 0.001). The final visual outcome remained good despite the recurrences. Good recovery despite a significant axonal loss may be due to redundancy in the visual system or cortical plasticity.[55]

## Management of Atypical Optic Neuritis

Failure of spontaneous visual recovery in the absence of any other cause, relapse on stopping the corticosteroids should arouse suspicion of non-MS causes of the optic neuritis. In the presence of the fact that the prevalence of NMO and infective and other inflammatory causes is much high in this part of the world, it becomes important to thoroughly investigate these patients. The list is exhaustive and is discussed in the ‘Diagnosis’ section. For acute attack of NMO, high-dose methylprednisolone and plasma exchange has been shown to be effective.[56,57] For the prevention of further attacks, immunosuppressive therapy in the form of oral azathioprine or mycophenolate mofetil with or without low-dose prednisolone or rituximab should be considered. Similarly, optic neuritis secondary to infective and inflammatory cause would require the treatment of the associated specific condition.

## Conclusion

Optic neuritis is essentially a clinical diagnosis. So a careful history and examination are essential in differentiating a typical optic neuritis from atypical, steroid-responsive and infective causes. If atypical features are present, urgent further investigations are indicated to find out causes of inflammation of optic nerve head and treat them accordingly. It is more important to pick up cases which do not spontaneously improve or show progressive deterioration, as one needs to rule out compressive or infective lesions and treat them urgently. Most cases of idiopathic or demyelinating optic neuritis have a good visual recovery irrespective of administration of intravenous steroids. Intravenous dexamethasone can be given to patients of acute optic neuritis as a substitute for methylprednisolone due to its easy availability and low cost. Treatment is particularly required in cases with recurrent attacks, in patients with a history or evidence of other neurological involvement, in atypical cases and in acute optic neuritis in children. Patients showing one or more lesions characteristic of MS or any other demyelinating lesion are promptly referred to a neurologist after explaining the advantages of immunomodulator therapy and kept on a long-term close follow-up. At present, due to the low prevalence of MS reported in Asia and the possible low likelihood of MS after a single episode of optic neuritis, the role of interferons does not appear to be justified following an isolated attack of typical optic neuritis.

While the majority of patients of typical optic neuritis recover without treatment, a small percentage of patients continue to have low vision despite intravenous steroid therapy. Research insights into the pathophysiology of optic neuritis may help develop more effective therapies in the future, probably by exploring the role of remyelination or adaptive neuroplasticity in the process of recovery.
